# Two-Dimensional Cs_3_Sb_2_I_9−x_Cl_x_ Film with (201) Preferred Orientation for Efficient Perovskite Solar Cells

**DOI:** 10.3390/ma15082883

**Published:** 2022-04-14

**Authors:** Jihong Li, Yongao Lv, Huifang Han, Jia Xu, Jianxi Yao

**Affiliations:** 1 State Key Laboratory of Alternate Electrical Power System with Renewable Energy Sources, North China Electric Power University, Beijing 102206, China; lijihong2413@gmail.com (J.L.); lvyongao@163.com (Y.L.); huifanghan@ncepu.edu.cn (H.H.); xujia@ncepu.edu.cn (J.X.); 2 Beijing Key Laboratory of Energy Safety and Clean Utilization, North China Electric Power University, Beijing 102206, China

**Keywords:** lead-free, Sb-perovskite, Cs_3_Sb_2_I_9−x_Cl_x_, solar cells

## Abstract

All-inorganic Sb-perovskite has become a promising material for solar cell applications owing to its air stability and nontoxic lead-free constitution. However, the poor morphology and unexpected (001) orientation of Sb-based perovskite films strongly hinder the improvement of efficiency. In this work, two-dimensional Cs_3_Sb_2_Cl_x_I_9−x_ with (201) preferred orientation has been successfully fabricated by introducing thiourea (TU) to the precursor solution. The presence of the C=S functional group in TU regulates the crystallization dynamics of Cs_3_Sb_2_I_9−x_Cl_x_ films and generates the (201) preferred orientation of Cs_3_Sb_2_Cl_x_I_9−x_ films, which could effectively improve the carrier transport and film morphology. As a result, the Cs_3_Sb_2_I_9−x_Cl_x_ perovskite solar cells (PSCs) delivered a power conversion efficiency (PCE) of 2.22%. Moreover, after being stored in nitrogen at room temperature for 60 days, the devices retained above 87.69% of their original efficiency. This work demonstrates a potential pathway to achieve high-efficiency Sb-based PSCs.

## 1. Introduction

The power conversion efficiency (PCE) of perovskite solar cells (PSCs) has achieved 25.7% owing to the optimal optoelectronic performance [1]. However, the toxicity of water-soluble lead remains a major deterrent to the application of Pb-based PSCs, posing a threat to soil and underground water resources. [2]. Therefore, several environmentally friendly elements (Sn, Bi) have been used to replace Pb [3,4]. So far, Sn-PSCs, Sb-PSCs, and Bi-PSCs have demonstrated the highest PCEs of 14.6%, 3.43%, and 3.2%, respectively [5,6,7]. Nevertheless, the main issue is that the Sn-based perovskite is liable to oxidize to Sn^4+^ in air or an inert environment [8].

Recently, all-inorganic Cs_3_Sb_2_I_9_ perovskites have also attracted significant interest because of their high absorption coefficient (>10^5^ cm^−1^) and excellent moisture and thermal stability [9,10,11]. In general, two main crystal structures exist for Cs_3_Sb_2_I_9_, namely, the zero-dimensional (dimer) and two-dimensional (layered) phases [12]. The 0 D phase has inherent problems, including poor carrier transport and an indirect bandgap greater than 2.2 eV, which are detrimental to the PCE [13]. Jiang et al. proved in theory and practice that replacing iodide with chloride in the Cs_3_Sb_2_I_9_ lattice can effectively suppress the formation of undesirable 0 D phases [9]. Peng et al. also reported that the replacement of a fraction of iodine with chlorine could obtain high-quality 2D Cs_3_Sb_2_Cl_x_I_9−x_ films with a PCE of 2.15% [14]. Umar et al. fabricated 2D Cs_3_Sb_2_I_9_ via a HCl-assisted solution method, but the rapid crystallization rate produced smaller grain sizes (~50 nm), limiting the efficiency to 1.21% [13]. To decrease the rate of perovskite crystallization and increase the grain size, N-Methyl-2-pyrrolidone (NMP), thiourea (TU), and bis (trifluoromethane) sulfonimide lithium (LiTFSI) have been adopted as an additive to form complexes with Sb^3+^, which could retard the perovskite formation process through an intramolecular exchange [7,15,16]. Generally, the preferential (001) plane of Cs_3_Sb_2_I_9_ is parallel to the substrate, which is a big obstacle preventing the improvement of the device performance. As we know, it is expected that the perovskite crystal grows vertical to the electrodes to enable an efficient charge transport up and down [17].

Therefore, modulation of the Cs_3_Sb_2_I_9_ crystal orientation to facilitate carrier transport was an issue of concern for Cs_3_Sb_2_I_9_ solar cells. Singh et al. found that introducing the coordination molecule indacenodithiophene-based organic acceptor (ITIC) into Cs_3_Sb_2_I_9_ films could effectively enhance the (201) orientation and achieve a PCE of 3.25% [10]. According to these studies, either Cl^−^ or coordination molecules could improve the crystallization of Sb-based perovskite and enhance the photovoltaic performance.

In this work, high quality Cs_3_Sb_2_I_9−x_Cl_x_ films with the preferential orientation of the (201) plane have been successfully fabricated by introducing TU to regulate perovskite crystallization. It was determined that the C=S group in TU can combine with Sb^3+^ to form a complex in the perovskite precursor, which significantly retards the crystallization. X-ray photoelectron spectra (XPS) verified that the TU could be eventually removed from the films, which had no effect on the component of the perovskite films. Based on the density functional theory (DFT) calculations, TU has a much lower adsorption energy (−0.22 eV) on the perovskite (003) crystalline plane than it does on the (201) plane (−0.36 eV). The perovskite (003) crystalline plane with a lower adsorption energy grows much more quickly than the (201) crystalline plane. In accordance with the Bravais–Riedel–Donnay–Harker laws, there is a tendency for crystalline planes growing quickly to decrease or even vanish [18]. As a result, high-quality Cs_3_Sb_2_I_9−x_Cl_x_ films with the (201) preferential orientation and a large grain size have been successfully obtained by optimizing the amount of TU in the precursors. The PCE has been promoted to 2.22%, with a notable multiplied *J_SC_* of 6.77 mA cm^−2^, *V_OC_* of 0.65 V, and FF of 50.3%. Moreover, the devices retained 87.69% of its initial efficiency after being stored in a nitrogen environment at room temperature for 60 days.

## 2. Experimental

### 2.1. Materials

SbI_3_ (98% metals basis, Sigma–Aldrich, St. Louis, MO, USA), SbCl_3_ (Sigma–Aldrich, St. Louis, MO, USA), Bis-(trifluoromethane) sulfonimide lithium salt (Xi’an Polymer Light Technology Corp, Xi’an, China), Thiourea (TU) (Sigma–Aldrich, St. Louis, MO, USA), tert-butylpyridine (tBP) (Xi’an Polymer Light Technology Corp, Xi’an, China), CsI (Xi’an Polymer Light Technology Corp, Xi’an, China), spiro-OMeTAD (99.7% Borun Chemicals, Zhejiang, China), DMF (Alfa-Aesar, Ward Hill, MA, USA), Isopropanol (J&K Scientific Co., Ltd., Beijing, China), and FK209-cobalt (III)-TFSI (MaterWinChemicals, Shanghai, China) were used as received.

### 2.2. Device Fabrication

The FTO substrates were cleaned with deionized water and ethanol for 20 min in an ultrasonic bath and then a UV–ozone treatment was performed for 30 min. The compact TiO_2_ and mp–TiO_2_ layers were deposited according to our previous method [19]. The precursor solution was prepared by dissolving 1 M CsI, 0.23 M SbCl_3_, and 0.02 M SbI_3_ in a 1 mL mixed of DMSO and DMF (3:1, *v*/*v*) and stirred at 70 °C for 12 h. Then the solution was spin–coated on FTO/c–TiO_2_/mp–TiO_2_ substrates at 3000 rpm for 30 s. After spin coating, the films annealed at 70 °C for 10 min to remove the solvents, followed by a SbI_3_ vapour annealing at 250 °C for 15 min. The SbI_3_ vapour was prepared by a 30 wt% SbI_3_ DMF solution (5 µL) dropping to the corner of the petri dish during annealing. For the TU additive sample, the desired amount of TU was added into the precursor and the above-mentioned film-formation protocol was followed. After that, the hole transporting layer and electrode were deposited according to our previous method [19].

### 2.3. Device Characterization

UV–vis spectra were acquired with the UV–2450 (Shimadzu, Kyoto, Japan) from 400 nm to 900 nm. XRD patterns were obtained via an X-ray diffractometer (XRD, SmartLab, Rigaku, Tokyo, Japan) with Cu–Kα radiation (1.5418) (5°–55°, 4° min^−1^). The morphologies and roughness were measured with the SU8010 SEM (Hitachi, Chiyoda City, Japan, 3.0 kV, 10100 nA) and 5500 AFM (Agilent Technologies, Santa Clara, CA, USA), respectively. Steady–state photoluminescence (PL) spectra were collected with a Fluorolog–322 (Horiba, Edison, NJ, USA). Time-resolved photoluminescence (TRPL) spectra were measured with the FLS1000 (Edinburgh Instruments, Livingston, UK). The FTIR spectrum was acquired with the Excalibur 3100 (Varian, Palo Alto, CA, USA). X–ray spectra (XPS) were acquired with the ESCALab250Xi, Thermo Fisher Scientific, Waltham, MA, USA. Impedance spectroscopy was measured via a Zahner electrochemical workstation (Zahner, Kronach, Germany) with a bias potential of 0.50 V in the dark with the frequency ranging from 100 Hz to 1 MHz. The *J*–*V* curves were tested via a Keithley 2400 source-meter under AM 1.5G 100 mW cm^−2^ from a sunlight simulator (XES–300T1, SAN–EI Electric, Tokyo, Japan). The capacitance-voltage (C–V) measurements was performed using a Zahner electrochemical workstation in the dark at a frequency of 10 kHz and the AC amplitude was 10 mV. The incident photon–to–electron conversion efficiency (IPCE) was recorded by a QE-R measurement system (Enli Technology, Kaohsiung City, Taiwan).

### 2.4. DFT Calculations

The first–principles calculations were done with VASP (Vienna Ab–initio Simulation Package) [20]. In order to optimize the Cs_3_Sb_2_I_9_ structure, a generalized gradient approximation (GGA) with the Perdew–Burke–Ernzerhof (PBE) function was employed [21]. A projector augmented wave (PAW) was used to describe the wave function of the core region, while a linear combination of plane waves with 550 eV cut-off energy was used to describe the valence wave function. Gamma point was used to sample the reciprocal space. During geometry optimization, 10^−5^ eV and −0.1 eV/ang were used as the convergence criteria for SCF and force, respectively.

## 3. Results and Discussion

**Figure 1:**
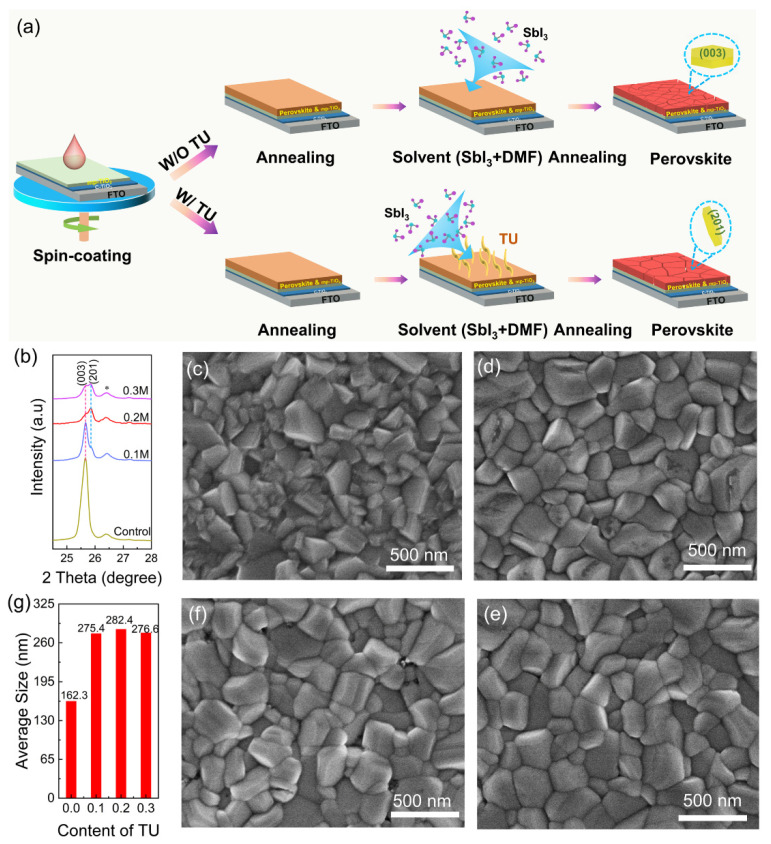
(**a**) Illustration of the Sb-based perovskite film fabrication procedure. (**b**) XRD patterns of the prepared Cs_3_Sb_2_I_9−x_Cl_x_ film with various amounts of TU. The XRD peak of “*” symbols belong to the TiO_2_/FTO substrates. (**c**–**f**) SEM of the control, 0.1, 0.2 M TU, and 0.3 M additive Cs_3_Sb_2_I_9−x_Cl_x_ films. (**g**) The grain size statistical distribution of the control, 0.1M, 0.2M, and 0.3M TU additive Cs_3_Sb_2_I_9−x_Cl_x_ films.

Figure 1a illustrates the preparation processes of the Sb-based perovskite thin film. For the W/O TU case, the films were first annealed at 70 °C for 10 min, ensuring the organic solvents could be removed completely, and then annealed in SbI_3_ vapour at 250 °C for 15 min. Finally, the perovskite film was obtained. The high SbI_3_ vapour pressure was considerable at 250 °C and effectively prevented the Cs_3_Sb_2_I_9−x_Cl_x_ film from losing SbI_3_ [22,23]. For the W/TU case, TU decomposed during the SbI_3_ vapour annealing process. Figure S1a illustrates the XRD patterns of Cs_3_Sb_2_I_9−x_Cl_x_ films containing various amounts of TU additives. The diffraction peaks at 8.7°, 17.2°, 21.0°, 25.7°, 25.9°, 29.9°, 42.7°, and 42.9° were ascribed to the (001), (002), (102), (003), (201), (022), (204), and (220) planes of the 2D Cs_3_Sb_2_I_9−x_Cl_x_ crystal phase, respectively [24]. Figure 1b shows an enlarged view of the (003) and (201) peaks in Figure S1a. In Figure 1b and Figure S1a, the relative intensities of (001), (002), and (003) peaks decrease with increasing amounts of TU. According to previous reports, the orientation of the (001), (002), and (003) planes parallel to the substrate leads to carrier transport anisotropy, which strongly limits the devices performance [10]. In addition, it can be observed in Figure 1b that the intensity of the (201) diffraction peak increases with an increasing amount of TU. The calculated data of the XRD parameters are shown in Table S1. The FWHM of the (201) plane for control, 0.1 M, 0.2 M, and 0.3 M TU additive Cs_3_Sb_2_I_9−x_Cl_x_ films was 1.516, 0.464, 0.436, and 0.454, respectively. The crystallite size of the Cs_3_Sb_2_I_9−x_Cl_x_ films was calculated by the Scherrer equation [25], the corresponding crystallite size for control, 0.1 M, 0.2 M, and 0.3 M TU was 5.621, 18.393, 19.356, and 18.795, respectively. The dislocation density of the (201) peak for control, 0.1 M, 0.2 M, and 0.3 M TU additive Cs_3_Sb_2_I_9−x_Cl_x_ films was 31.88, 2.96, 2.61, and 2.85, respectively. The lattice constants for control, 0.1 M, 0.2 M, and 0.3 M are also listed in Table S1. It was found that the 0.2 M TU additive enhanced the crystallinity of the Cs_3_Sb_2_I_9−x_Cl_x_ films. To further understand the influence of TU on the crystal orientation of the Cs_3_Sb_2_I_9−x_Cl_x_, we calculated the peak intensity ratio (201)/(003) for the Cs_3_Sb_2_I_9−x_Cl_x_ films, and the results are shown in Figure S1b and Table S2. For the control sample, the peak intensity ratio of the (201)/(003) diffraction peaks is 0, since the (201) peak is barely detectable. The peak intensity ratios of the (201)/(003) diffraction peaks are 0.34, 2.01, and 1.25 for the 0.1 M, 0.2 M, and 0.3 M TU additives, respectively. The maximum value of (201)/(003) for the 0.2 M TU additive Cs_3_Sb_2_Cl_x_I_9−x_ films exhibits the preferred (201) growth orientation of the Cs_3_Sb_2_I_9−x_Cl_x_ crystals (Figure S1b). Figure 1c–f shows the scanning electron microscopy (SEM) images of the control, 0.1 M, 0.2 M, and 0.3 M TU additive Cs_3_Sb_2_I_9−x_Cl_x_ films. The film without TU (Figure 1c) shows a rough and irregular morphology with small grain sizes. In contrast, films with the TU additive (Figure 1d–f) exhibit a smooth and compact film with a large grain. In particular, the 0.2 M TU additive film demonstrates the largest grain sizes and high compactness. According to Lewis acid–base adduction matching rules, sulphur is a kind of soft base that can form a stable coordination with Sb^3+^, further modulating perovskite nucleation and crystallization [26,27]. However, further increasing the addition of TU to 0.3 M results in cavities on the surface of the perovskite, which could be due to the gas generated from TU thermal decomposition (CH_4_N_2_S = H_2_S + CH_2_N) [19]. Meanwhile, the statistical distribution of grain sizes in the Cs_3_Sb_2_I_9−x_Cl_x_ films with the addition of different amounts of TU obtained from top-view SEM images using nano measurer software is shown in Figure 1g. Apparently, the average grain size of perovskite increases significantly after the addition of TU. It is worth noting that the 0.2 M TU additive Cs_3_Sb_2_Cl_x_I_9−x_ films show a maximum average grain size of 282.4 nm, which is much larger than that of the control film of 162.3 nm (Figure 1g). The increase in the grain sizes and reduction in the boundary areas could reduce charge carrier trapping [28]. In Figure S2a,b, the film roughness of the control and 0.2 M TU additive Cs_3_Sb_2_I_9−x_Cl_x_ films were 72.6 nm and 21.9 nm, respectively, indicating that the 0.2 M TU additive Cs_3_Sb_2_I_9−x_Cl_x_ films have small surface roughness. Figure S2c,d show the Kelvin probe force microscope (KPFM) of the control and the 0.2 M TU additive Cs_3_Sb_2_I_9−x_Cl_x_ films, respectively. The 0.2 M TU additive Cs_3_Sb_2_I_9−x_Cl_x_ films surface exhibited a higher electronic chemical potential than that of the control film, resulting from a low surface trap density and high carrier concentration [29,30]. Additionally, to further explore the dispersion of elements in the perovskite film, energy-dispersive spectroscopy (EDS) mapping was carried out to characterize the 0.2 M TU additive Cs_3_Sb_2_I_9−x_Cl_x_ films (Figure S3). As shown in Figure S3, the films demonstrate a uniform distribution of the Cs, Sb, I, and Cl elements. Meanwhile, no S was found in the film.

**Figure 2:**
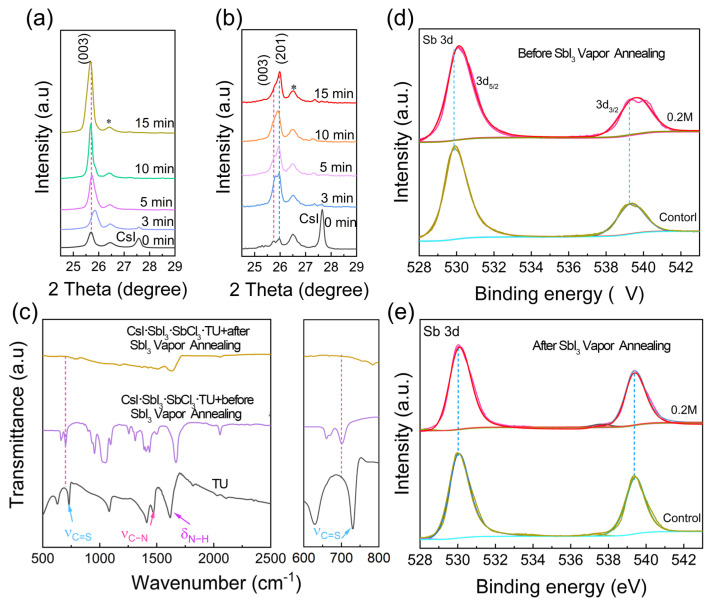
XRD diffraction patterns of (**a**) the control and (**b**) 0.2 M TU additive Cs_3_Sb_2_I_9−x_Cl_x_ films under different SbI_3_ vapour annealing time. The annealing time was assigned 0 min, 3 min, 5 min, 10 min, and 15 min, respectively. The XRD peak of “*” symbols belong to the TiO_2_/FTO substrates. (**c**) FTIR spectra of TU, CsI·SbI_3_·SbCl_3_·TU before SbI_3_ vapour annealing, and CsI·SbI_3_·SbCl_3_·TU after SbI_3_ vapour annealing. (**d**,**e**) XPS for Sb 3d core level spectra in control and 0.2 M TU additive Cs_3_Sb_2_Cl_x_I_9−x_ films before and after SbI_3_ vapour annealing, respectively.

The XRD patterns of the control and the 0.2 M TU additive Cs_3_Sb_2_I_9−x_Cl_x_ films with different SbI_3_ vapour annealing times are shown in Figure S4a,b. Figure 2a shows an enlarged view of the (003) peak in Figure S4a. As shown in Figure 2a, the (003) peak first shifted to larger angles and then to smaller angles with increasing SbI_3_ vapour annealing time. The shift is especially obvious for 3 min in SbI_3_ vapour annealing. The (201) diffraction peak could not be seen in Figure 2a. When the annealing time was further extended from 5 min to 10 min, no further changes in the peak position were observed. It was obvious that a large amount of chlorine first entered the Cs_3_Sb_2_I_9−x_Cl_x_ lattice, and then it was eliminated from the Cs_3_Sb_2_I_9−x_Cl_x_ lattice during SbI_3_ vapour annealing due to the small ionic radius of Cl^−^. Furthermore, the intensity of the (003) peak increased as the SbI_3_ vapour annealing time increased, indicating the enhanced crystallinity of Cs_3_Sb_2_I_9−x_Cl_x_ films. The (003) and (201) peaks in Figure S4b are enlarged in Figure 2b. In contrast to the result shown in Figure 2a, an obvious (201) diffraction peak appears in Figure 2b. The (201) diffraction peak initially increases and then remains unchanged in the SbI_3_ vapour annealed for 10 min (Figure 2b). The intensity of the (003) diffraction peak initially increased and then decreased as annealing in SbI_3_ increased. These results clearly demonstrate that TU strongly affects the orientation of the Cs_3_Sb_2_I_9−x_Cl_x_ films. In the presence of TU, the intermediate phase CsI·SbI_3_·SbCl_3_·TU would be formed in the precursor solution. Figure S5 shows Fourier-transform infrared (FTIR) spectra of the TU, CsI·SbI_3_·SbCl_3_·TU before SbI_3_ vapour annealing, and CsI·SbI_3_·SbCl_3_·TU after SbI_3_ vapour annealing. Figure 2c shows an enlarged view of infrared peaks in Figure S5. The peak at a wavenumber of 730 cm^−1^ corresponds to the stretching vibration of C=S in TU, which is shifted to 700 cm^−1^ for CsI·SbI_3_·SbCl_3_·TU before SbI_3_ vapour annealing [31]. The shift of the C=S stretching vibration frequency was ascribed to the decrease of the bond between carbon and sulphur upon the adduct interaction of TU and CsI·SbI_3_·SbCl_3_ [32]. Therefore, TU was involved in the CsI·SbI_3_·SbCl_3_·TU formation process. After the SbI_3_ vapour annealing, the intermediate phase changed to Cs_3_Sb_2_I_9−x_Cl_x_ perovskite phase, and TU was thermally decomposed, as shown in Figure 2c.

To further investigate the elemental characteristics of the control and 0.2 M TU additive Cs_3_Sb_2_I_9−x_Cl_x_ films, XPS (X-ray photoelectron spectroscopy) was performed. Figure S6 shows XPS survey spectra of the 0.2 M TU additive Cs_3_Sb_2_I_9−x_Cl_x_ films before and after SbI_3_ vapour annealing. It clearly indicates the presence of Cs, Sb, I, Cl, and S in the films before SbI_3_ vapour annealing. Figure 2d,e shows the XPS results of Sb 3d peaks for the control and 0.2 M TU additive Cs_3_Sb_2_I_9−x_Cl_x_ films before and after SbI_3_ vapour annealing. For the control and 0.2 M TU additive Cs_3_Sb_2_I_9−x_Cl_x_ films before SbI_3_ vapour annealing, the Sb 3d5/2 peaks are located at 529.88 eV and 530.1 eV, respectively, which indicates a strong intermolecular interaction between Sb^3+^ and TU [24]. Moreover, as shown in Figure 2e, the Sb 3d5/2 peaks had no obvious change for the control and 0.2 M TU additive Cs_3_Sb_2_I_9−x_Cl_x_ film after SbI_3_ vapour annealing. This result also indicates no residual TU in the final perovskite film. To further verify this point, we study the high-resolution S 2p and N 1s XPS spectra for the 0.2 M TU additive Cs_3_Sb_2_I_9−x_Cl_x_ films after different SbI_3_ vapour annealing times. As shown in Figure S7a, the characteristic peak of N 1s located at 399.7 eV tends to decrease and disappear after 10 min of SbI_3_ vapour annealing. In Figure S7b, the peak at 162.01 eV corresponds to S 2p of the TU. Meanwhile, no S 2p signals were detected in the TU additive perovskite film after 5 min of SbI_3_ vapour annealing. Therefore, we deduced that TU was removed from the perovskite film during SbI_3_ vapour annealing [33].

**Figure 3:**
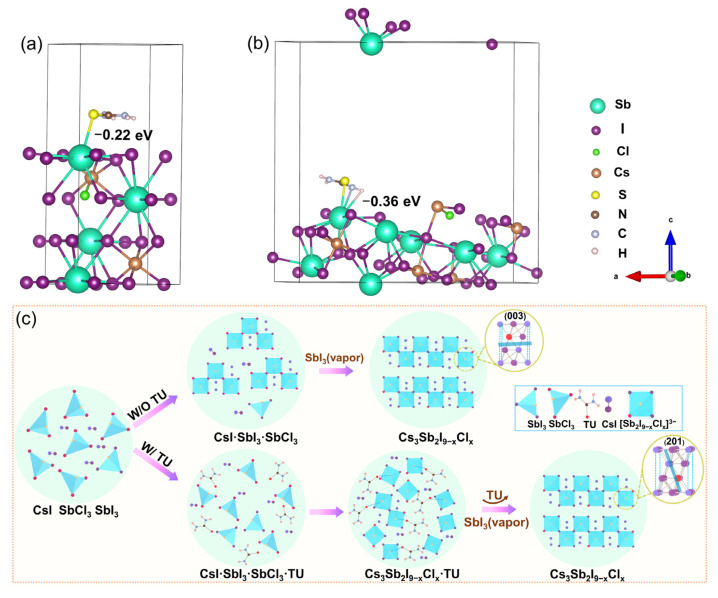
Atomic structure of optimized Cs_3_Sb_2_I_8_Cl (**a**) (001) and (**b**) (201) surface with TU molecule. (**c**) Illustration of the crystallization process of Cs_3_Sb_2_I_9−x_Cl_x_ perovskite film.

Figure 3a,b shows the atomic structure of the optimized Cs_3_Sb_2_I_8_Cl (001) and (201) surfaces with the TU molecule. In Figure 3a,b, the TU molecule was placed on Cs_3_Sb_2_I_8_Cl (003) and (201) planes, in which the C=S groups anchor at the central Sb ion. The theoretical results based on DFT reveal that TU is adsorbed strongly on the (201) plane with an adsorption energy of −0.36 eV, which is much larger than that on the (003) plane of −0.22 eV. In addition, the large adsorption energy indicates that TU molecules interact strongly with the (201) oriented Cs_3_Sb_2_I_9−x_Cl_x_ plane. According to previous studies, the (003) plane with a lower adsorption energy with TU grows much faster than the (201) plane and eventually disappears, which agrees with the XRD results [34]. Figure 3c provides a schematic diagram of the crystallization process of the Cs_3_Sb_2_I_9−x_Cl_x_ film. For the W/O TU case, the Cs_3_Sb_2_I_9−x_Cl_x_ films crystallize rapidly during annealing. For the W/TU case, the intermediate phase CsI·SbI_3_·SbCl_3_·TU could effectively delay the rapid nucleation during the annealing to obtain dense and smooth Cs_3_Sb_2_I_9−x_Cl_x_ films along with TU decomposition [35].

**Figure 4:**
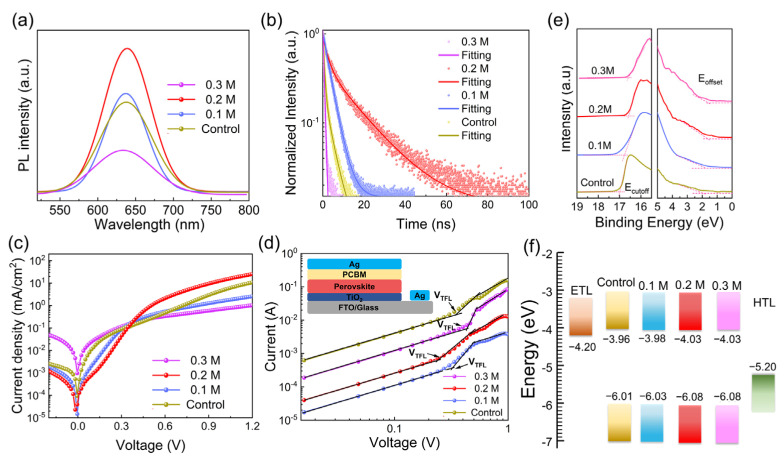
(**a**) Steady state PL spectra and (**b**) Time-resolved PL spectra of the control and different amounts of TU additive Cs_3_Sb_2_I_9−x_Cl_x_ perovskite films. (**c**) Dark *J*–*V* curves of control and TU additive Cs_3_Sb_2_I_9−x_Cl_x_ perovskite films. (**d**) Electron-only devices for measuring the trap-state density of control and TU additive Cs_3_Sb_2_I_9−x_Cl_x_ perovskite films. (**e**) UPS spectra corresponding to the valence band (VB) and cutoff regions of the control and different amounts of TU additive Cs_3_Sb_2_I_9−x_Cl_x_ perovskite films. (**f**) Energy diagrams of the control and different amounts of TU additive Cs_3_Sb_2_I_9−x_Cl_x_ perovskite films.

Figure S8a shows the UV–vis absorption spectrum for Cs_3_Sb_2_I_9−x_Cl_x_ films containing a different content of TU additive. Tauc plots for the assessed band gap value are shown in Figure S8b. Figure S8b shows that the control and TU additive perovskite film absorption edge were all located at 605 nm (2.05 eV), similar to previous reports [24]. Steady-state photoluminescence (PL) and time-resolved photoluminescence (TRPL) decay measurements were carried out to explore the trap states and charge recombination dynamics in control and different amounts of TU additive Cs_3_Sb_2_I_9−x_Cl_x_ films, and its structure was glass substrate/Cs_3_Sb_2_I_9−x_Cl_x_ film. As shown in Figure 4a, it is clear that the emission peaks are located at around 625 nm, conforming to the previous report [10]. The PL peak intensity of the Cs_3_Sb_2_I_9−x_Cl_x_ film shows a trend of increasing first and then decreasing with the increases of TU from 0.1 M to 0.3 M. The 0.2 M TU additive film exhibits the highest PL peak intensity, which indicates that the nonradiative recombination was suppressed [36]. When the amount of TU was 0.3 M, the PL peak intensity became weakened as a result of the increase in the nonradiative recombination and defect density. This might be caused by the presence of cavities on the 0.3 M TU additive film due to the gas generated from the thermal decomposition of TU (CH_4_N_2_S = H_2_S + CH_2_N) [19]. Meanwhile, the 0.3 M TU additive could lead to impurities in the film, which would increase the nonradiative recombination. Fewer traps in the Cs_3_Sb_2_I_9−x_Cl_x_ film with the 0.2 M TU additive were further confirmed by TRPL, as shown in Figure 4b and Table S3. The average decay lifetime was prolonged from 2.16 ns (control) to 15.5 ns (0.2 M TU additive), indicating that the nonradiative recombination could be reduced owing to the low defect states of large-grained Cs_3_Sb_2_I_9−x_Cl_x_ films. The TRPL results were in good agreement with the steady-state PL results. Therefore, the smooth surface, large grain size, and high crystallinity of Cs_3_Sb_2_I_9−x_Cl_x_ films played a crucial role in reducing the defects states and further suppressing the nonradiative recombination.

Moreover, to evaluate the charge transfer properties of the devices, the electron state density (N_trap_) was calculated. We also measured *J*–*V* curves of devices in the dark (Figure 4c). The 0.2 M TU additive Cs_3_Sb_2_I_9−x_Cl_x_ films had a much lower leakage current than the other devices, which might be associated with the increase of grain size and reduction of defects in the film [37]. Space for charge limited current (SCLC) also was used to estimate defect properties of the devices and the results are shown in Figure 4c and Table S4. The trap state densities were calculated on the basis of the formula: [38] N_t_ = 2ε_0_εV_TFL_/qL^2^ (q is the elemental charge, ε_0_ is the vacuum permittivity, ε is the relative dielectric constant and L is the thickness of the Cs_3_Sb_2_I_9−x_Cl_x_ film). For the 0.2 M TU additive Cs_3_Sb_2_I_9−x_Cl_x_ films, the lower trap-filled limit voltage (V_TFL_) was 0.23 V for electron-only, which indicated the trap density had an impressive reduction. Ultraviolet photoelectron spectroscopy (UPS) results in Figure 4e show that the control, 0.1 M TU, 0.2 M TU, and 0.3 M TU additive Cs_3_Sb_2_I_9−x_Cl_x_ films have valence band maxima at −6.01 eV, −6.03 eV, −6.08 and −6.08 eV, respectively. Figure 4f shows a schematic energy level diagram of the materials used in the device. Figure S9 shows the band energy diagrams for the control, 0.1 M, 0.2 M and 0.3M TU additive Cs_3_Sb_2_I_9−x_Cl_x_ PSCs. The energy-level alignment of the conduction band minimum between Cs_3_Sb_2_I_9−x_Cl_x_ and TiO_2_ was improved through the addition of TU into Cs_3_Sb_2_I_9−x_Cl_x_ film. The VB/CB edge of the absorber shifted downwards upon the addition of TU. These downshifts in VB/CB benefited the electric charge transport and improved the PCE [39].

**Figure 5:**
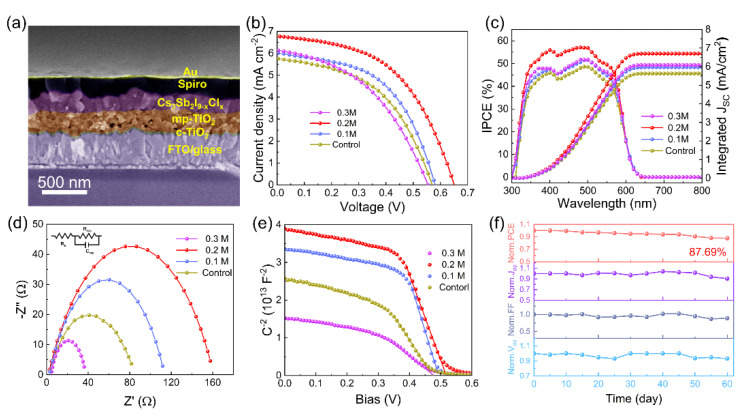
(**a**) Cross−section SEM image of the device. Comparison of the fabricated Cs_3_Sb_2_I_9−x_Cl_x_ films with different amounts of TU: (**b**) *J*–*V* curves; (**c**) EQE and integrated current density; (**d**) Nyquist plots; (**e**) Mott–Schottky curve. (**f**) Long−term stability of non−encapsulated PSCs in N_2_ environment conditions.

The cross-sectional SEM image of the device based on a 0.2 M TU additive Cs_3_Sb_2_I_9−x_Cl_x_ perovskite film is shown in Figure 5a, which has a thickness of cp−TiO_2_, mp−TiO_2_, Cs_3_Sb_2_I_9−x_Cl_x_, Spiro-OMeTAD, and Au layers for 20 nm, 200 nm, and 290 nm, 90 nm, and 65 nm, respectively. Figure S10a,b show the cross-sectional SEM images of control and 0.2 M TU additive Cs_3_Sb_2_I_9−x_Cl_x_ films (290 nm), respectively. It is apparent that the 0.2 M TU additive Cs_3_Sb_2_I_9−x_Cl_x_ film grains exhibit a uniform structure from bottom to top. The uniform and dense perovskite grains will enhance the carrier transport in the device [40]. The *J*–*V* curves for devices fabricated with Cs_3_Sb_2_I_9−x_Cl_x_ films with different amounts of TU are presented in Figure 5b, and the specific performance parameters are shown in Table S5. In Figure 5b, as the TU content increases, the short–circuit density (*J_SC_*) improves, and the 0.2 M TU additive Cs_3_Sb_2_I_9−x_Cl_x_ films devices obtain the highest efficiency of 2.22% under the 1–sun condition, which is attributed to the preferred orientation, smooth surface, and high crystallinity of perovskite films resulting in an improved carrier transport. The comparison of the efficiency with representative studies of Cs_3_Sb_2_I_9−x_Cl_x_ PSCs summarized in Table S6, which clearly illustrates that our research produces a high *J_SC_* for PSCs while maintaining an excellent PCE [10,13,14,24,41]. The Cs_3_Sb_2_I_9−x_Cl_x_ films with 0.3 M TU additive exhibited a poor photoelectric performance as a result of the appearance of a pinhole in the film. The external quantum efficiency (EQE) spectrum is shown in Figure 5c. The increases of integrated *J_SC_* from 5.75 mA cm^−2^ to 6.77 mA cm^−2^ upon the addition of 0.2 M TU was in accordance with the measured *J_SC_* from the *J*–*V* plots. The data of the fabricated PSCs are counted in Figure S11. These statistical parameters clearly reveal that the 0.2 M TU additive could efficiently enhance the photovoltaic performance of the PSCs. The Nyquist plots and fitting lines measured under ambient–air dark conditions are shown in Figure 5d. The inset gives an equivalent circuit. Table S7 lists the fitting parameters. Notably, the 0.2 M TU additive Cs_3_Sb_2_I_9−x_Cl_x_ PSCs device shows the largest charge recombination resistance (R_rec_) (178.1) compared to the control device (99.1). The series resistance of the 0.2 M TU additive of the Cs_3_Sb_2_I_9−x_Cl_x_ PSCs device exhibits a lower value (7.2 Ω) than that of control (9.4 Ω) due to the improved crystal orientation and morphology of perovskite film, demonstrating the boosting of charge transfer and the enhancing of the fill factor (FF) value. Capacitance-voltage (C−V) was carried out to explain the kinetics of the charge recombination and the increment of voltage values. The built−in potentials can be extracted in accordance with the Mott–Schottky Equation (1): [19].

(1) $$\frac{1}{C^{2}}=\frac{2}{\varepsilon_{0}\varepsilon eA^{2}N_{A}}\left(V_{\mathrm{bi}}-\;V\right)$$

where C is the capacitance, V is the applied voltage, V_bi_ is the built–in potential, A is the device area, ε is the relative permittivity, ε_0_ is the vacuum permittivity, and N_A_ is the carrier concentration. As shown in Figure 5e, the V_bi_ of 0.2 M TU additive Cs_3_Sb_2_I_9−x_Cl_x_ PSCs was 0.65 V, which was higher than that of the control, 0.1 M TU additive, and 0.3 M TU additive devices 0.49 V, 0.46 V, and 0.45 V, respectively. The increasing V_bi_ benefitted the separation of the photogenerated carriers, which suppressed the electron-hole recombination and the [42]. The long-term stability of the 0.2 M TU additive Cs_3_Sb_2_I_9−x_Cl_x_ solar cells in N_2_ environment conditions was observed (Figure 5f). The PCE of the optimal device retained approximately 87.69% of the initial value after 60 days.

## 4. Conclusions

In summary, we successfully fabricated a high-quality Cs_3_Sb_2_I_9−x_Cl_x_ film using a TU additive. TU promoted Cs_3_Sb_2_I_9−x_Cl_x_ oriented crystallization. The induced Cs_3_Sb_2_I_9−x_Cl_x_ perovskites prefered to grow in the (201) orientation, which is favourable for carrier transport. Moreover, TU also reduced the crystallization rate of Cs_3_Sb_2_I_9−x_Cl_x_ perovskites and improved the morphology of the film. The regulated crystallization orientation and morphology efficiently assisted in reducing the trap state density, restraining nonradiative recombination, and elongating the carrier lifetime. As a result, the 0.2 M TU additive devices displayed an optimal PCE of 2.22%, along with a prolonged lifetime of 87.69% for the initial PCE after 60 days in N_2_ environment. This work shows a good prospect for modulating the crystallization process and orientation for high-performance Cs_3_Sb_2_I_9−x_Cl_x_ perovskite photovoltaic devices.

## Data Availability

The data that support the findings of this study are available from the corresponding author upon reasonable request.

## Supplementary Materials

The following supporting information can be downloaded at: https://www.mdpi.com/article/10.3390/ma15082883/s1, Figure S1: (a) XRD patterns of the prepared Cs_3_Sb_2_I_9−x_Cl_x_ films with different amounts of TU additive. The “*” symbols represent the signals of the TiO_2_/FTO substrates. (b) The intensity ratio of I_(201)_/I_(003)_ for Cs_3_Sb_2_I_9−x_Cl_x_ films with different amounts of TU additive; Figure S2. (a,b) AFM; (c,d) KPFM of the control and 0.2 M TU additive Cs_3_Sb_2_I_9−x_Cl_x_ films; Figure S3. SEM images of 0.2 M TU additive Cs_3_Sb_2_I_9−x_Cl_x_ films, corresponding element distribution maps of Cs, Sb, I, Cl, and S; Figure S4. XRD diffraction patterns of (a) the control and (b) 0.2 M TU additive Cs_3_Sb_2_I_9−x_Cl_x_ films under different SbI_3_ vapor annealing time. The annealing time was assigned 0, 3, 5, 10, and 15 min, respectively. The “*” symbols represent the signals of the TiO_2_/FTO substrates; Figure S5. FTIR spectra of TU, CsI·SbI_3_·SbCl_3_·TU before SbI_3_ vapour annealing, and CsI·SbI_3_·SbCl_3_·TU after SbI_3_ vapour annealing; Figure S6. (a) XPS survey spectra of the 0.2 M TU additive Cs_3_Sb_2_I_9−x_Cl_x_ films before and after SbI_3_ vapour annealing; Figure S7. (a,b) XPS results of the core-level peak for N 1s and S 2p were obtained from the 0.2 M TU additive Cs_3_Sb_2_I_9−x_Cl_x_ films under different SbI_3_ vapor annealing times; Figure S8. (a) UV-visible and (b) Tauc plots of Cs_3_Sb_2_I_9−x_Cl_x_ thin films with different amounts of TU additive; Figure S9. Schematic of the band energy diagrams for the control, 0.1 M, 0.2 M and 0.3 M TU additive Cs_3_Sb_2_I_9−x_Cl_x_ PSCs; Figure S10. (a,b) Cross-sectional image of the control and 0.2 M TU additive Cs_3_Sb_2_I_9−x_Cl_x_ films; Figure S11. (a–d) Comparison of histograms of photovoltaic parameters for the perovskite solar cells based on control and 0.2M TU additive condition. (Data from 20 cells were used for the histogram); Table S1: XRD parameters of FWHM, crystallite size (D = 0.9λ/β cosθ), dislocation densities (δ = n/D^2^, n is a factor which is almost equal to unity for minimum dislocation density), and lattice constants for the control and different amounts of TU Cs_3_Sb_2_I_9__−x_Cl_x_ films; Table S2. Intensity ratios of (201)/(003) planes of Cs_3_Sb_2_I_9−x_Cl_x_ films with different amounts of TU; Table S3. TRPL decay parameters for the control and 0.2 M TU additive Cs_3_Sb_2_I_9−x_Cl_x_ films; Table S4. The electron trap density of Cs_3_Sb_2_I_9−x_Cl_x_ films with different amounts of TU additive; Table S5. Photovoltaic parameters of the perovskite solar cells fabricated with different amounts of TU additive; Table S6. A summary of the device performance of Cs_3_Sb_2_I_9−x_Cl_x_ solar cells; Table S7 Parameters fitted from electrochemical impedance spectra of the device based on Cs_3_Sb_2_I_9−x_Cl_x_ films with different amounts of TU additive.
