# Supplementary material for: Two-Dimensional Cs3Sb2I9−xClx Film with (201) Preferred Orientation for Efficient Perovskite Solar Cells
Source: Materials (Basel). 2022 Apr 14;15(8):2883. doi: 10.3390/ma15082883 (PMC9032497; doi:10.3390/ma15082883)
Supplement: Supplementary file 1 [file materials-15-02883-s001.zip › materials-1651445-supplementary.pdf]

materials

MDPI

Supplementary

Two-dimensional $Cs_3Sb_2I_{9-x}Cl_x$ film with (201) preferred orientation for efficient perovskite solar cells

Jihong Li [1,2], Yongao Lv [1,2], Huifang Han [1,2], Jia Xu [1,2], and Jianxi Yao [1,2,*]

[1] State Key Laboratory of Alternate Electrical Power System with Renewable Energy Sources, North China Electric Power University, Beijing 102206, China; lijihong2413@gmail.com; lvyongao@163.com; huifanghan@ncepu.edu.cn; xujia@ncepu.edu.cn

[2] Beijing Key Laboratory of Energy Safety and Clean Utilization, North China Electric Power University, Beijing 102206, China

* Correspondence: jianxiyao@ncepu.edu.cn

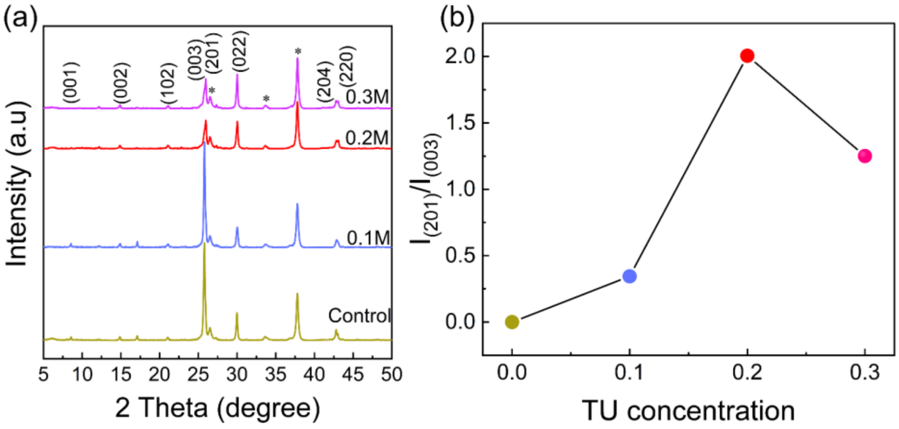

Figure S1. (a) XRD patterns of the prepared $Cs_3Sb_2I_{9-x}Cl_x$ films with different amounts of TU additive. The "*" symbols represent the signals of the TiO_2/FTO substrates. (b) The intensity ratio of $I_{(201)}/I_{(003)}$ for $Cs_3Sb_2I_{9-x}Cl_x$ films with different amounts of TU additive.

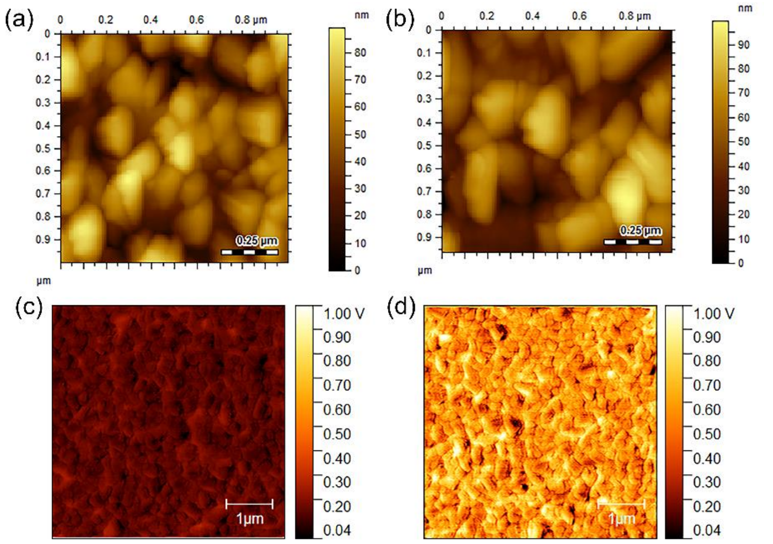

Figure S2. (a, b) AFM; (c, d) KPFM of the control and 0.2M TU additive $Cs_3Sb_2I_{9-x}Cl_x$ films.

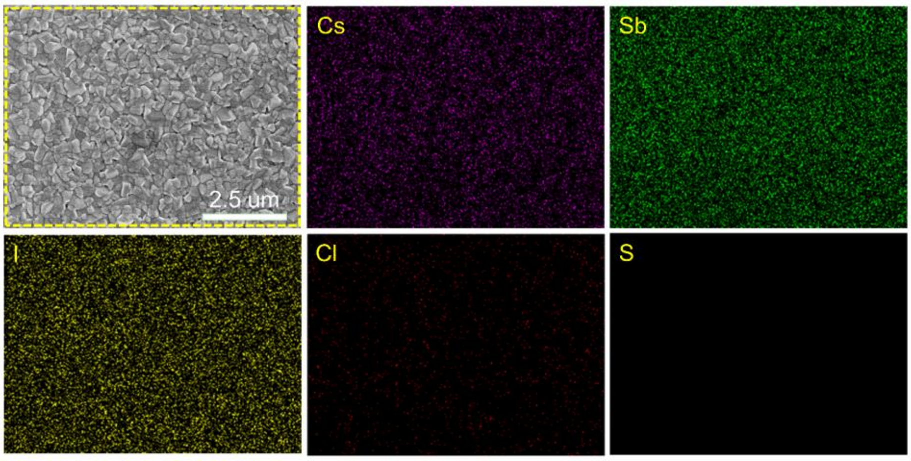

Figure S3. SEM images of 0.2M TU additive $Cs_3Sb_2I_{9-x}Cl_x$ films, corresponding element distribution maps of Cs, Sb, I, Cl, and S.

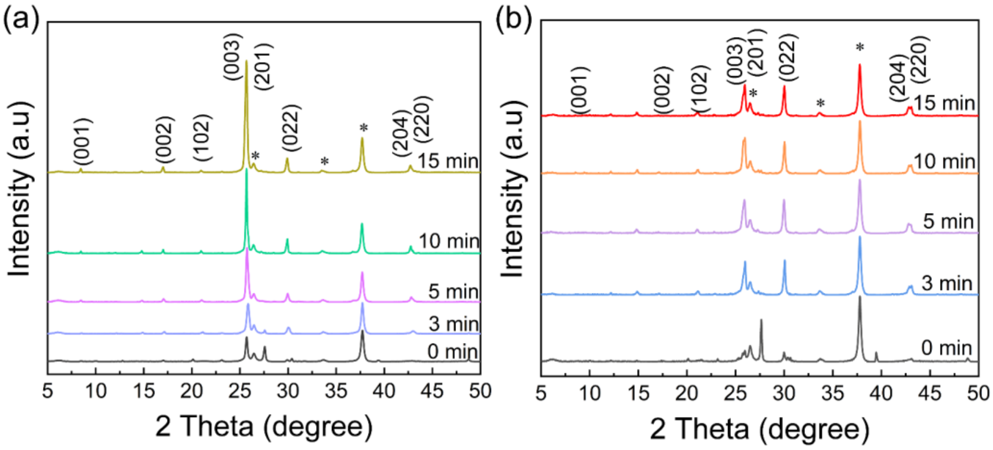

Figure S4. XRD diffraction patterns of (a) the control and (b) 0.2M TU additive $Cs_3Sb_2I_{9-x}Cl_x$ films under different SbI_3 vapor annealing time. The annealing time was assigned 0, 3, 5, 10, and 15 min, respectively. The "*" symbols represent the signals of the TiO_2/FTO substrates.

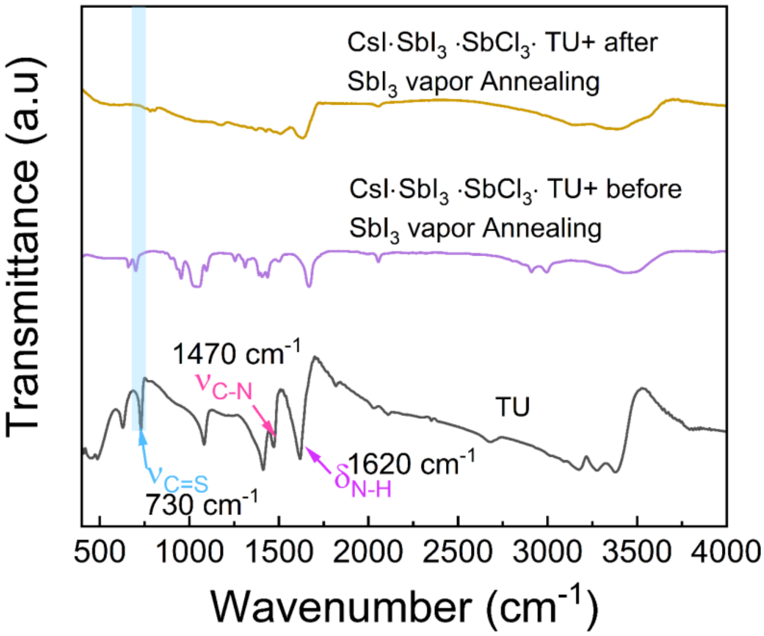

Figure S5. FTIR spectra of TU, CsI·SbI_3·$SbCl_3$·TU before SbI_3 vapour annealing, and CsI·SbI_3·$SbCl_3$·TU after SbI_3 vapour annealing.

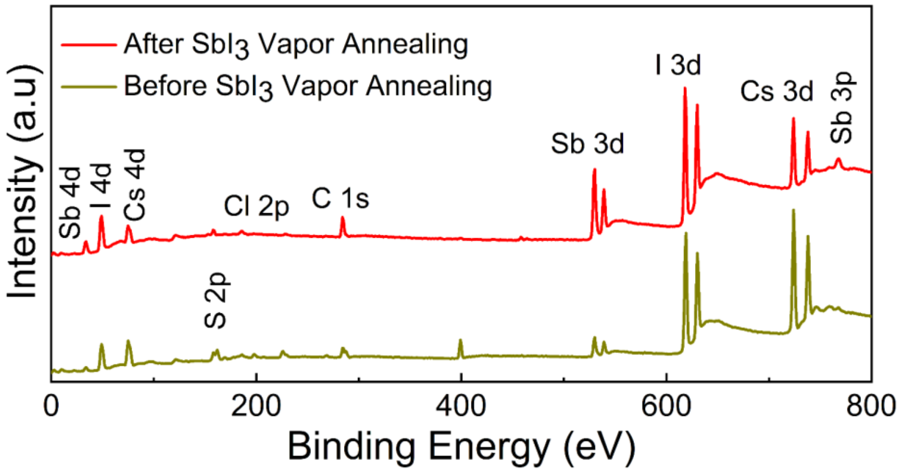

Figure S6. (a) XPS survey spectra of the 0.2 M TU additive $Cs_3Sb_2I_{9-x}Cl_x$ films before and after SbI_3 vapour annealing.

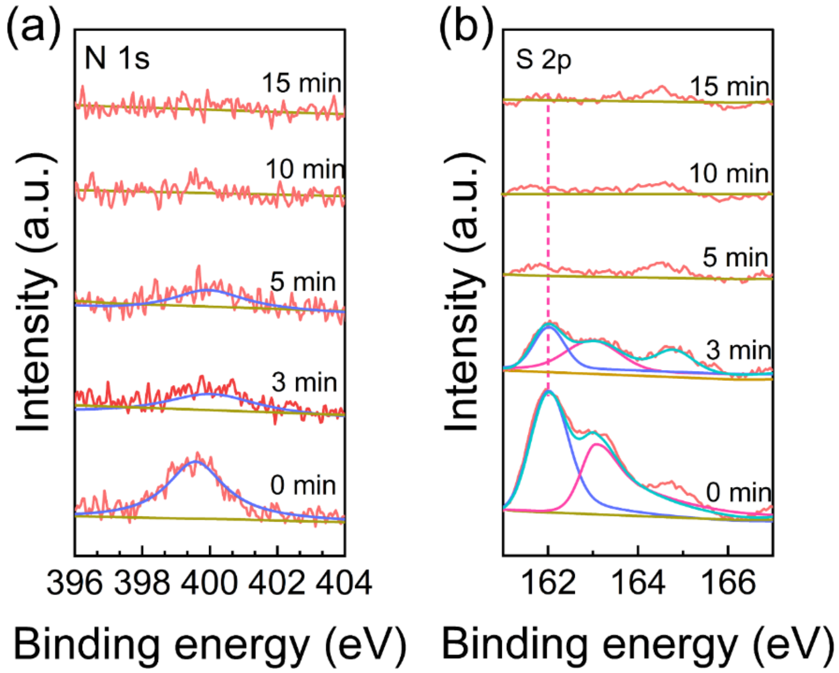

Figure S7. (a-b) XPS results of the core-level peak for N 1s and S 2p were obtained from the 0.2M TU additive $Cs_3Sb_2I_{9-x}Cl_x$ films under different SbI_3 vapor annealing times.

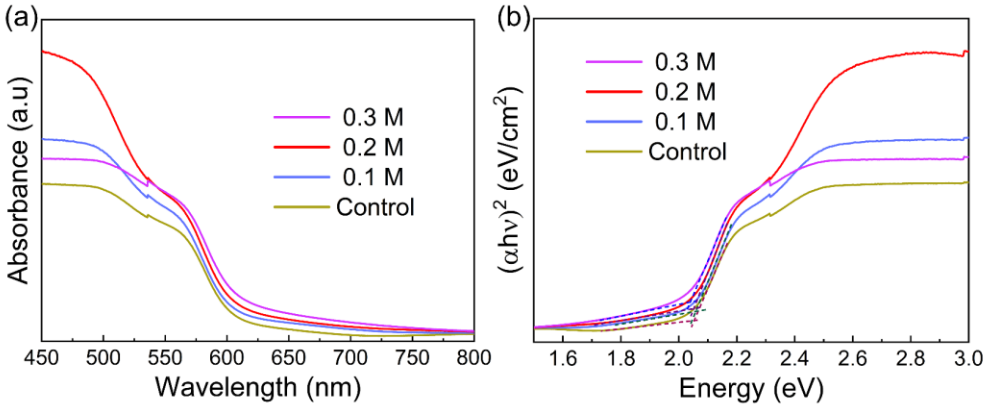

Figure S8. (a) UV-visible and (b) Tauc plots of $Cs_3Sb_2I_{9-x}Cl_x$ thin films with different amounts of TU additive.

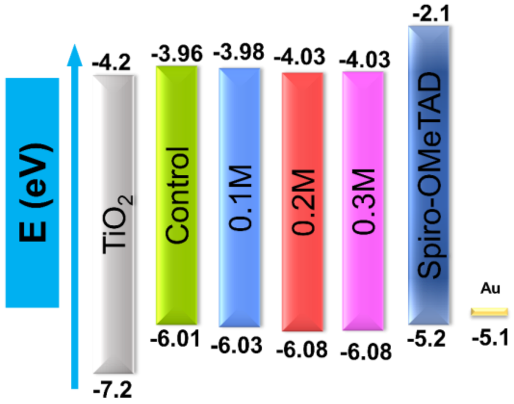

Figure S9. Schematic of the band energy diagrams for the control, 0.1 M, 0.2 M and 0.3M TU additive $Cs_3Sb_2I_{9-x}Cl_x$ PSCs.

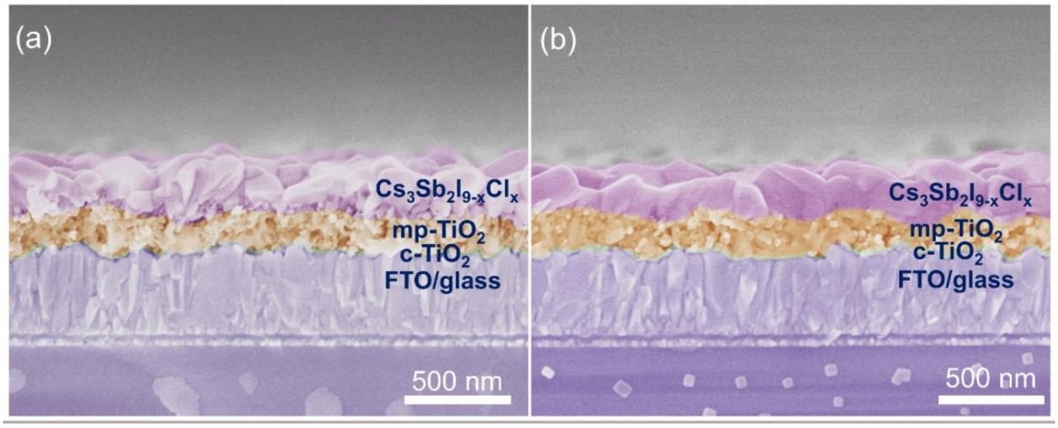

Figure S10. (a-b) Cross-sectional image of the control and 0.2M TU additive $Cs_3Sb_2I_{9-x}Cl_x$ films.

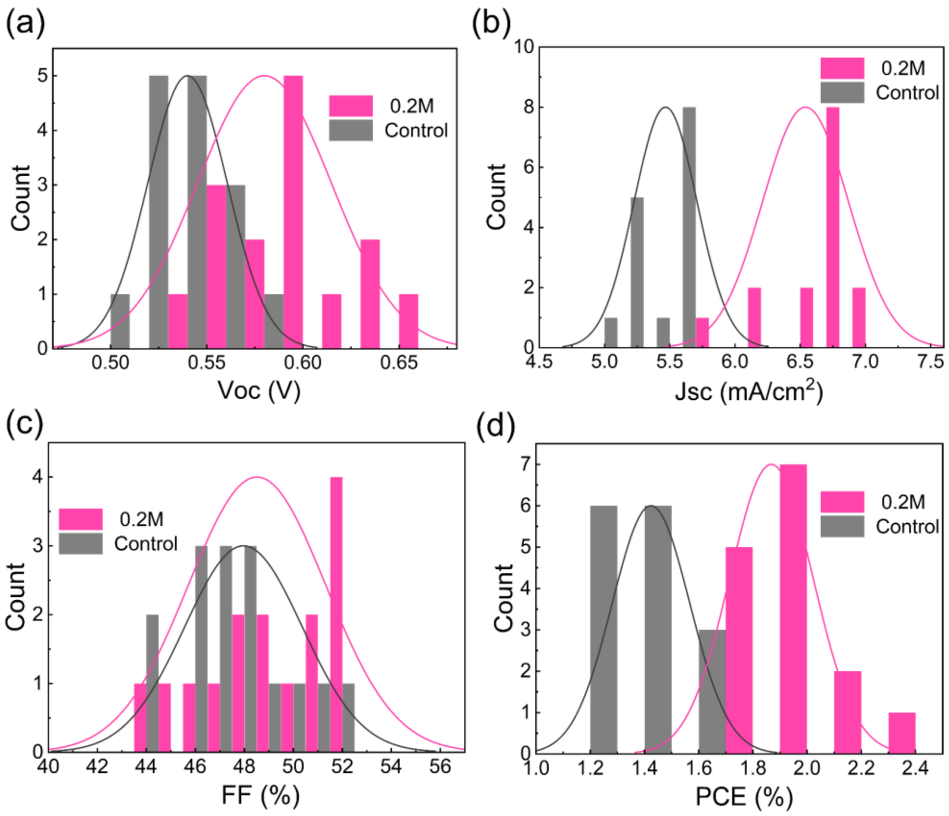

Figure S11. (a-d) Comparison of histograms of photovoltaic parameters for the perovskite solar cells based on control and 0.2M TU additive condition. (Data from 20 cells were used for the histogram).

Table S1. XRD parameters of FWHM, crystallite size (D=0.9λ/β·cosθ), dislocation densities ($\delta = n/D^2$, n is a factor which almost equals to unity for minimum dislocation density), and lattice constants for the control and different amount of TU $Cs_3Sb_2I_{9-x}Cl_x$ films.

TU concentration	FWHM (°)	crystallite size (nm)	dislocation densities (10^{11}) cm^{-2}	Lattice constant (Å)	
				a=b	c
Control	1.516	5.621	31.88	8.422	10.386
0.1 M	0.464	18.383	2.96	8.412	10.362
0.2 M	0.436	19.356	2.61	8.429	10.356
0.3 M	0.454	18.795	2.83	8.431	10.363

Table S2. Intensity ratios of (201)/(003) planes of $Cs_3Sb_2I_{9-x}Cl_x$ films with different amount of TU.

Device	**(201)/(003)**
Control	0.00
0.1 M	0.34
0.2 M	2.01
0.3 M	1.25

Table S3. TRPL decay parameters for the control and 0.2M TU additive $Cs_3Sb_2I_{9-x}Cl_x$ films.

Device	τ_1 (ns)	τ_2 (ns)	τ_{ave} (ns)
Control	0.654	3.788	2.1628
0.1 M	3.659	4.024	3.7770
0.2 M	5.000	21.155	15.4836

0.3 M	0.459	5.685	1.8872

Table S4. The electron trap density of $Cs_3Sb_2I_{9-x}Cl_x$ films with different amounts of TU additive.

**Device**	V_{TFL}	electron trap density
Control	0.35 V	3.1x10^{15} cm^{-3}
0.1 M	0.32 V	2.8x10^{15} cm^{-3}
0.2 M	0.23 V	2.0x10^{15} cm^{-3}
0.3 M	0.43 V	3.7x10^{16} cm^{-3}

Table S5. Photovoltaic parameters of the perovskite solar cells fabricated with different amounts of TU additive.

Device	V_{OC} (V)	J_{SC} (mA cm^{-2})	FF (%)	PCE (%)
Control	0.56	5.75	48.7	1.59
0.1 M	0.58	6.02	51.6	1.80
0.2 M	0.65	6.77	50.3	2.22
0.3 M	0.55	6.13	43.5	1.48

Table S6. A summary of the device performance of $Cs_3Sb_2I_{9-x}Cl_x$ solar cells.

Structure	Voc (V)	Jsc (mA cm^2)	PCE (%)	Ref
ITO/PEDOT: PSS/ITIC-treated $Cs_3Sb_2I_9$/ITIC/Ca/Ag	0.91	6.45	3.25	Chem. Eng. J, 2021, 419,129424.
ITO/PEDOT:PSS/$Cs_3Sb_2Cl_xI_{9-x}$/PCBM/Al	0.72	6.39	2.2	Sol. RRL, 2021, 5, 2000422.
FTO/TiO_2/$Cs_3Sb_2Cl_3I_6$/poly-TPD/Au	0.60	6.46	2.15	Appl. Mater. Today. 2021, 9, 15301.
FTO/TiO_2/$Cs_3Sb_2Cl_3I_6$/poly-TPD/Au	0.61	3.55	1.21	Adv. Opt. Mater, 2019, 7, 1801368
FTO/TiO_2/$Cs_3Sb_2I_9$/P3HT/Au	0.80	5.40	2.48	Sustain. Energy Fuels. 2022, 6, 217-222.
**FTO/bl-TiO_2/TU-treated $Cs_3Sb_2Cl_xI_{9-x}$ /Sipro/Au**	**0.65**	**6.77**	**2.22**	**This work**

Table S7 Parameters fitted from electrochemical impedance spectra of the device based on $Cs_3Sb_2I_{9-x}Cl_x$ films with different amounts of TU additive.

Device	R_s/Ω·cm^2	R_{rec}/Ω·cm^2
Control	9.4	99.1
0.1 M	8.8	130.2
0.2 M	7.2	178.1
0.3M	12.3	36.2
